# Comparative benefits and harms of perioperative interventions to prevent chronic pain after orthopedic surgery: a systematic review and network meta-analysis of randomized trials

**DOI:** 10.1186/s13643-024-02528-x

**Published:** 2024-04-26

**Authors:** Mohammed Al-Asadi, Kian Torabiardakani, Andrea J. Darzi, Ian Gilron, Maura Marcucci, James S. Khan, Luis E. Chaparro, Brittany N. Rosenbloom, Rachel J. Couban, Andrew Thomas, Jason W. Busse, Behnam Sadeghirad

**Affiliations:** 1 Faculty of Health Sciences, McMaster University, Hamilton, ON Canada; 2 Department of Anesthesia, McMaster University, Hamilton, ON L8S 4K1 Canada; 3 Department of Health Research Methods, Evidence, and Impact (HEI), McMaster University, Hamilton, ON Canada; 4 Michael G. DeGroote National Pain Centre, McMaster University, Hamilton, ON Canada; 5 Departments of Anesthesiology and Perioperative Medicine, Queen’s University, Kingston, Canada; 6 Departments of Biomedical and Molecular Sciences, Queen’s University, Kingston, Canada; 7 Centre for Neuroscience Studies, Queen’s University, Kingston, Canada; 8 School of Policy Studies, Queen’s University, Kingston, ON Canada; 9 Department of Anesthesiology and Pain Medicine, University of Toronto, Toronto, ON Canada; 10 Department of Anesthesia, Grand River Hospital, Kitchener, ON Canada; 11 Toronto Academic Pain Medicine Institute, Women’s College Hospital, Toronto, ON Canada; 12 Canadian Armed Forces Health Services Centre, Edmonton, AB Canada; 13 Clinical Epidemiology and Research Centre (CERC), Department of Biomedical Sciences, Humanitas University & IRCCS Humanitas Research Hospital, Milan, Italy

**Keywords:** Chronic postsurgical pain, Comparative effectiveness, Systematic review, Orthopedic surgery, Musculoskeletal surgery

## Abstract

**Background:**

Chronic postsurgical pain (CPSP) is common following musculoskeletal and orthopedic surgeries and is associated with impairment and reduced quality of life. Several interventions have been proposed to reduce CPSP; however, there remains uncertainty regarding which, if any, are most effective. We will perform a systematic review and network meta-analysis of randomised trials to assess the comparative benefits and harms of perioperative pharmacological and psychological interventions directed at preventing chronic pain after musculoskeletal and orthopedic surgeries.

**Methods:**

We will search MEDLINE, Embase, PsycINFO, CINAHL, and the Cochrane Central Register of Controlled Trials from inception to present, without language restrictions. We will include randomised controlled trials that as follows: (1) enrolled adult patients undergoing musculoskeletal or orthopedic surgeries; (2) randomized them to any pharmacological or psychological interventions, or their combination directed at reducing CPSP, placebo, or usual care; and (3) assessed pain at 3 months or more after surgery. Screening for eligible trials, data extraction, and risk-of-bias assessment using revised Cochrane risk-of-bias tool (RoB 2.0) will be performed in duplicate and independently. Our main outcome of interest will be the proportion of surgical patients reporting any pain at ≥ 3 months after surgery. We will also collect data on other patient important outcomes, including pain severity, physical functioning, emotional functioning, dropout rate due to treatment-related adverse event, and overall dropout rate. We will perform a frequentist random-effects network meta-analysis to determine the relative treatment effects. When possible, the modifying effect of sex, surgery type and duration, anesthesia type, and veteran status on the effectiveness of interventions will be investigated using network meta-regression. We will use the GRADE approach to assess the certainty evidence and categorize interventions from most to least beneficial using GRADE minimally contextualised approach.

**Discussion:**

This network meta-analysis will assess the comparative effectiveness of pharmacological and psychological interventions directed at preventing CPSP after orthopedic surgery. Our findings will inform clinical decision-making and identify promising interventions for future research.

**Systematic review registration:**

PROSPERO CRD42023432503.

**Supplementary Information:**

The online version contains supplementary material available at 10.1186/s13643-024-02528-x.

## Background

Chronic pain is a prevalent condition, and its burden is estimated to be large and growing [1, 2]. Approximately, one in four patients undergoing surgery experiences chronic pain [3]. Chronic postsurgical pain (CPSP) is defined as pain at or near the site of surgery that persists for more than 3 months after the procedure, which is not otherwise explained by a preexisting condition or infection [4, 5]. The prevalence of CPSP varies by type of surgery but is particularly common after orthopedic surgeries with estimates ranging from 5% for laminectomy and spinal fusion to 85% following limb amputation [4, 6].

Chronic pain after surgery is associated with reduced psychological and physical health, and quality of life [7]. Often, patients with CPSP require additional treatments and healthcare costs, with some cost estimates exceeding US $1 million per patient over their lifetime [8, 9]. Over 310 million surgical procedures are performed globally each year, of which 40 million are orthopedic [10]. The number of patients suffering from chronic pain after musculoskeletal and orthopedic surgeries is therefore considerable.

Several systematic reviews have explored the effectiveness of treatments for the prevention of CPSP including psychotherapy [11], gabapentinoids [12, 13], antidepressants [14], ketamine [15], opioids [16], nonsteroidal anti-inflammatory drugs (NSAIDs) [17], muscle relaxants [18], acetaminophen [19], and corticosteroids [17, 20]. Most existing trials focus on treatment effects of interventions relative to placebo or usual care; however, comparisons between active therapies are more important when deciding between several competing interventions [21]. Additionally, the multiplicity of interventions available most often leads to separate fragmented meta-analyses, instead of a single big picture identifying superior interventions for optimized clinical decision making [22, 23]. Network meta-analysis (NMA) allows for the estimation of comparative effectiveness among treatment options and combines direct and indirect evidence for each comparison which improves the precision of effect estimates [22, 23]. This is especially helpful when considering interventions informed by trials with small numbers of participants, which is often the case for treatments directed at management of CPSP [17].

There is very limited evidence available on relative benefits and harms of different pharmacotherapies and psychological perioperative interventions for preventing CPSP. The occurrence of CPSP depends on type and approach of surgery [4, 24]. There is also considerable variability in risk factors of CPSP across different surgical procedures [4, 24, 25]. To limit heterogeneity in prognostic factors and relative treatment effects, our review will focus on trials of CPSP after musculoskeletal and orthopedic surgeries. Thus, our objective is to perform a comprehensive review to summarize the evidence from existing randomised controlled trials and assess the relative effects of perioperative interventions to prevent chronic pain after musculoskeletal and orthopedic surgeries.

## Methods

### Registration and reporting

We reported our protocol according to the Preferred Reporting Items for Systematic Reviews and Meta-Analysis Protocols (PRISMA-P) guideline [26] and registered it in PROSPERO (CRD42023432503). We will report our findings in an open-access journal following PRISMA-NMA guideline [27].

### Eligibility criteria

This review will include randomized trials with any design (parallel, crossover, or cluster randomized). Eligible population will be adult patients (≥ 18 years old) undergoing any type of musculoskeletal or orthopedic surgeries. The eligible interventions include any perioperative pharmacotherapy, any individual-based (therapy delivered to a single individual) or group-based (therapy delivered to a group of individuals treated at the same time) perioperative psychological interventions (e.g., online or in-person [cognitive] behavior therapy, psychoeducation, counseling or supportive therapy, guided imagery therapy, pain coping skills training, relaxation or mindfulness-based therapy, and acceptance and commitment therapy), or their combination, aiming at preventing CPSP. Eligible comparisons include any of the interventions listed above or usual care, placebo, waitlist control, or no treatment. Our main outcome of interest is proportion of patients with CPSP (as defined by International Classification of Diseases, 11th revision (ICD-11) or earlier revisions depending on trial publication date). Other outcomes of interest for our review are proportion of patients with moderate-to-severe CPSP, pain severity, physical functioning or disability, emotional functioning, drop-out rate due to treatment-related adverse events, and overall drop-out rate. We will exclude studies that fail to report any of our outcomes of interest or those that only report overall drop-out rate without any effectiveness outcome.

Studies with patients who underwent a variety of surgical procedures will be included if outcomes are reported separately for musculoskeletal and/or orthopedic surgery patients. Examples of eligible surgical procedures for this review include joint arthroplasty and joint replacement surgeries (due to degenerative or inflammatory diseases, trauma, or overuse injuries), discectomy, fusion surgeries (cervical, lumbar, or joint fusion), spine surgeries, soft tissue repair surgeries (e.g., fasciotomy, muscle repair, reconstruction surgeries for tendons and ligaments), fracture fixation, and osteotomy. We will include multi-arm trials with non-eligible interventions when at least two of the trial arms are eligible.

We will exclude dose-finding trials or those that randomize patients to different dosages of the same drug without any other active comparison, usual care, placebo, no treatment, or waitlist control. We will consult with our clinical experts to determine if a trial used a suboptimal dose or if we need to consider splitting a drug node based on dose. In addition, we will exclude trials comparing psychological interventions or pharmacotherapy combined with other active interventions (e.g., physical therapy, exercise, manual therapy) unless co-interventions were provided to all study participants.

### Data sources and search strategy

An experienced medical librarian (R. J. C.) developed database-specific search strategies for MEDLINE, Embase, PsycINFO, CINAHL, and the Cochrane Central Register of Controlled Trials through OVID platform (see Additional file 1). We will perform our searches without publication status or language restrictions from inception to 01 July 2023 and will update them every 3 months until submission of our study for publication through automatic alert services from electronic databases [28]. We will screen the reference list of included trials as well as relevant reviews for additional eligible studies not captured through our searches.

### Study selection and data extraction

After removing duplicate records from our electronic database searches, pairs of trained reviewers will screen titles and abstracts independently to identify potentially eligible records using DistillerSR, an online systematic review software (Evidence Partners, Ottawa, Canada; https://www.distillersr.com). Subsequently, the same pairs of reviewers will review full reports independently to confirm eligibility. We will use standardized screening forms and will perform calibration exercises to improve reliability of the screening process. Any disagreements will be resolved through discussion or adjudication by a third reviewer.

Using a standardized form, pairs of reviewers will independently extract relevant information from all eligible studies after performing calibration exercises. We will contact study authors to confirm eligibility and to request missing information when required. Data items to be abstracted will include the following: (i) study characteristics (e.g., author’s name, year of publication, study design, country where participants were enrolled, and source of funding), (ii) patient characteristics (e.g., mean age of participants, proportion of female participants, mean body mass index, proportion of veterans, pain level before surgery, percentage of participants diagnosed with psychiatric disorders), (iii) details of surgical procedure (e.g., type and duration of surgery, anesthesia), (iv) details of intervention and comparisons (e.g., dose, duration, route and frequency of administration), and (v) information on outcomes of interest. All effectiveness outcomes will be extracted and analyzed at three follow-up times, 3 to 6 months, 6 to 12 months, and more than 1 year. For these timepoints, when data is available for more than one follow-up time (e.g., data reported every 1 month), we will extract data from the longest follow-up. The exception will be for drop-out rates, where we will abstract data at the longest follow-up time reported.

For data available only in graphical form, we will estimate the numerical values using a freely available online pixel counting tool, WebPlotDigitizer (https://automeris.io/WebPlotDigitizer). For crossover trials, if there is evidence of period effect, we will only extract data from the first period. For cluster trials, we will calculate effective sample size and apply an intracluster correlation coefficient to the number of events or sample mean for continuous outcomes, as suggested by the Cochrane handbook [29]. Any disagreements will be resolved through discussion or adjudication by a third reviewer.

### Risk-of-bias assessment

Pairs of reviewers, working independently, will assess the risk of bias for the effect of assignment to the intervention (i.e., the intention-to-treat effect) for each outcome of interest, in each trial, using the Cochrane risk-of-bias tool (RoB 2.0) [29, 30] which assesses whether studies are at high, low risk of bias, or have some concerns across the following domains: [1] bias from the randomization process, (2) bias due to deviations from the intended interventions, (3) bias due to missing outcome data, (4) bias in measurement of the outcome, and (5) bias in selection of the reported results. Studies will be rated as low overall risk of bias if all domains are at low risk of bias, as high risk of bias if one or more domains are rated at high risk of bias, and as having some concerns in all the other cases. We will use versions of RoB 2 that deal with additional issues that arise in trials with cluster randomised or crossover designs and will use the signalling questions from the archived version of RoB 2.0 embedded in an MS Excel tool for RoB assessments (available from: www.riskofbias.info/welcome/rob-2-0-tool). Any disagreements will be resolved through discussion or adjudication by a third reviewer. RoBvis tool (available from https://mcguinlu.shinyapps.io/robvis) will be used to present a visual summary of risk of bias assessments.

### Data synthesis and statistical analysis

For pain severity, physical functioning, and emotional functioning, we will calculate the weighted mean difference and associated 95% CI. We will use change from baseline instead of end-of-study scores, and if change scores are not reported, we will calculate them using the baseline and end-of-study values and the associated standard deviation (SD) using a correlation coefficient derived from other studies as described in the Cochrane handbook [29]. We will use the methods described by Weir et al. [31] to impute means and SDs when only median, (interquartile) range, and sample size are reported, and we are unable to acquire these details from trial authors. When studies use different measurement instruments that capture a common construct (e.g., pain), we will first transform all outcomes to a common instrument score using methods explained by Thorlund et al. [32] prior to meta-analysis.

We will assess the feasibility of performing NMA for each outcome by checking network connectivity, ensuring the availability of more trials than number of intervention nodes and having at least 10 trials in any network, and assessing the transitivity assumption using NMA-studio web application (https://www.nmastudioapp.com) [31]. We will consider the distribution of possible prognostic factors (proportion of female participants, mean body mass index, pain level before surgery, percentage of participants diagnosed with psychiatric disorders, and type of anesthesia) across treatment comparisons to investigate potential intransitivity. When it is not feasible to perform NMA, we will perform conventional pairwise meta-analysis using DerSimonian-Laird random-effects model for any comparison informed by at least two trials. When it is not possible or appropriate to pool results using meta-analysis, we will tabulate and narratively describe any relevant data. Network structure and decisions to lump or split interventions will be based on clinical expert opinion, comparative effects of interventions, and consideration of NMA assumptions (network connectivity, transitivity, and incoherence).

When appropriate to perform NMA, we first calculate direct effect estimates using DerSimonian-Laird random-effects model and will use Cochran’s Q statistic and *I*^2^ to determine statistical heterogeneity. For binary outcomes (proportion of patients with CPSP, proportion of patients with moderate-to-severe pain, and drop out rates), we will calculate the pooled relative risk (RR) and the associated 95% confidence interval (CI). We then will use NMA estimates and baseline risks derived from the median risk in placebo arms of included trials to calculate the risk difference (RD) and 95% CIs.

To perform NMA, a contrast-based random-effects model with a common heterogeneity estimate using the methodology of multivariate meta-analysis using “network” suite in Stata will be used for all outcomes [32–34]. The “design-by-treatment” model will be used to examine the coherence assumption at network level (global test of coherence). The side-splitting method will be used to assess local (loop-specific) incoherence in each closed network loop as the difference between direct and indirect evidence [32, 35]. If significant incoherence is identified within the network, we will investigate the network for the source(s) of incoherence and subsequently expand or exclude the node(s) introducing incoherence. We will create network diagram at the intervention level to visualize the available evidence and will present league table showing relative effect estimates for all interventions.

When at least 10 trials contribute to a meta-analysis of a direct comparison, we will assess small-study effects for that meta-analysis using Harbord’s test [36] for binary outcomes and Egger’s test [37] for continuous outcomes. To explore the impact of important prognostic factors on network estimates of effect, we will examine the following subgroups when feasible by running network meta-regression: (1) veteran vs nonveteran, (2) sex (male vs female), (3) anesthesia type (local, regional, general, or combination), (4) surgical procedure (major vs minor surgical procedures), and (5) risk of bias (low vs high risk). For all listed subgroups except risk of bias, when available, within-study data will be extracted and analyzed. Stata (StataCorp., Release 17.0, College Station, TX, USA) will be used for all data analyses. All comparisons will be two-tailed using a threshold *P*-value ≤ 0.05.

### Certainty of evidence assessment

We will follow the GRADE (Grading of Recommendations Assessment, Development and Evaluation) Working Group’s recommended approach to assess the certainty of evidence for each network estimate across different outcomes [38]. This assessment considers both direct and indirect comparisons. Initially, we will evaluate the certainty of direct estimates, based on conventional GRADE guidelines [39, 40] considering the risk of bias, inconsistency, indirectness, and small-study effects. We then will evaluate the certainty of indirect estimates with a focus on the dominant lowest-order loop. Network estimates will be used for precision assessment, and we will use null value as the threshold for imprecision. We may decide to rate the certainty further down if we observe unexplained incoherence between direct and indirect estimates of effect.

### GRADE minimally contextualized approach for treatment hierarchy

NMAs often report ranking probabilities and values for surface under the cumulative ranking curves (SUCRA) among competing interventions. However, this approach has important limitations; ranking probabilities and SUCRA values are largely associated with the magnitude of point estimates and ignore the associated precision and certainty of evidence [21, 23]. Thus, we will apply a minimally contextualized approach to develop a treatments hierarchy that takes into consideration the effect estimates from NMA and their certainty of evidence [41, 42].

It is uncommon for a NMA to identify a single intervention that is superior to all others. Therefore, in this approach, interventions are classified into categories from most to least effective. For each outcome, we will classify interventions as follows: group 1, termed “among the least effective,” which includes the reference intervention (e.g., placebo) and any intervention no more effective than the reference; group 2, termed “among the intermediate,” which includes interventions superior to the reference, but not superior to other interventions; and group 3, interventions that prove superior to at least one group 2 intervention, termed “among the most effective.” We will use the same approach for dropouts due to adverse events and all-cause dropouts but will create groups of interventions as follows: (1) no more harmful than the reference, (2) among the intermediate, and (3) among the most harmful. We will then divide interventions in all groups into two categories based on certainty of evidence: those supported by moderate or high certainty evidence and those supported by low or very low certainty evidence.

## Discussion

CPSP is a prevalent complication after orthopedic surgery with an important impact on patient function and quality of life. With a current trend and the rate of surgeries, it is not unexpected to see significant rise in the number of patients seeking care to manage CPSP. As such, there is a need for a high-quality review to summarize the evidence on the comparative effectiveness of available perioperative interventions aimed at reducing the development of chronic pain after musculoskeletal and orthopedic surgical procedures.

Most existing trials and current systematic reviews and meta-analyses assess the effectiveness of treatments relative to placebo or usual treatment. Our study will address this limitation by synthesizing novel information about the comparative benefits and harms of available treatments. There is considerable variability in incidence of CPSP across different surgical procedures. Besides the type and approach of surgery, perioperative factors impact the incidence of CPSP. We intend to limit the variability in prognostic factors and reduce the heterogeneity in baseline risks and relative effect of interventions by focusing on musculoskeletal and orthopedic surgeries. Our findings will provide valuable insights to guide optimal pain management strategies and will highlight gaps in current evidence in this area.

Our proposed review will have several strengths. First, our review is restricted to a set of procedures assumed to be associated with higher rate of CPSP, and by limiting the patient population, we had hope to have a more homogeneous set of studies. Second, we will assess the certainty of evidence supporting the effects of interventions using GRADE approach and will apply novel and transparent methods to determine treatment hierarchies. Our review will be limited by the quality of included studies, and the generalizability of our findings to non-orthopedic surgical procedures will be uncertain.

### Supplementary Information

**Additional file 1:** Search strategies for MEDLINE, Embase, PsycInfo, CINAHL, and the Cochrane Central Register of Controlled Trials.

## Data Availability

Not applicable.

## Abbreviations

CPSP: Chronic postsurgical pain
SD: Standard deviation
NSAID: Nonsteroidal anti-inflammatory drug
NMA: Network meta-analysis
RCT: Randomized controlled trial
SUCRA: Surface under the cumulative ranking curve
GRADE: Grading of Recommendations Assessment, Development and Evaluation
