# Supplementary material for: Comparative benefits and harms of perioperative interventions to prevent chronic pain after orthopedic surgery: a systematic review and network meta-analysis of randomized trials
Source: Syst Rev. 2024 Apr 26;13:114. doi: 10.1186/s13643-024-02528-x (PMC11046964; doi:10.1186/s13643-024-02528-x)
Supplement: Supplementary file 1 — Additional file 1: Search strategies for MEDLINE, Embase, PsycInfo, CINAHL, and the Cochrane Central Register of Controlled Trials. [file 13643_2024_2528_MOESM1_ESM.docx]

**Additional file 1: Summary of searches and strategies for MEDLINE, Embase, PsycInfo, CINAHL, and the Cochrane Central Register of Controlled Trials**

**Database: OVID Medline Epub Ahead of Print, In-Process & Other Non-Indexed Citations, Ovid MEDLINE(R) Daily and Ovid MEDLINE(R) 1946 to Present**

Search Strategy:

--------------------------------------------------------------------------------

1 exp Orthopedic procedures/ or (orthop?edic* adj2 (procedure* or surgery or surgical or surgeries)).mp. (372072)

2 (Acetabuloplasty or acetabuloplasties).mp. (475)

3 (((amputation or amputations) adj2 surgical) or (disarticulation or hemipelvectomy)).mp. (24939)

4 (Arthrodesis or arthrodeses or spinal fusion* or lumbar fusion* or cervical fusion* or spondylodeses or spondylodesis or spondylosyndeses or spondylosyndesis).mp. (51103)

5 (Arthroplasty or arthroplasties or (bone tunnel adj (enlargement or enlargements or widening))).mp. (108400)

6 (Arthroscopy or arthroscopic).mp. [mp=title, book title, abstract, original title, name of substance word, subject heading word, floating sub-heading word, keyword heading word, organism supplementary concept word, protocol supplementary concept word, rare disease supplementary concept word, unique identifier, synonyms, population supplementary concept word, anatomy supplementary concept word] (43214)

7 Bone lengthening.mp. (2668)

8 (Bone transplantation or bone grafting).mp. (40024)

9 (cementoplasty or cementoplasties or osteoplasties or osteoplasty).mp. (1244)

10 ((Collateral or cruciate) adj ligament reconstruction).mp. (12821)

11 (diskectomy or diskectomies or discectomy or discectomies).mp. (11623)

12 Fasciotomy/ or (fascietomy or fasciotomy).mp. (6003)

13 ((fracture or skeletal) adj (surgery or fixation*)).mp. (73085)

14 (Intervertebral adj (Disc or disk) adj (Chemolysis or chemolyses or nucleolyses or nucleolysis)).mp. (489)

15 (Laminectomy or laminectomies or laminotomies or laminotomy).mp. (17265)

16 (Laminoplasty or laminaplasties or laminaplasty or laminoplasties).mp. (2236)

17 Limb Salvage.mp. (10483)

18 (Osteotomy or osteotomies).mp. (51101)

19 (Synovectomy or synovectomies or synovium resection*).mp. (4333)

20 Tendon transfer*.mp. (5874)

21 (Tenodesis or tenodeses).mp. (2339)

22 (Tenotomy or ((heel cord or heel-cord or tendon) adj (release or releases or lengthening or lengthenings))).mp. (3705)

23 (vertebroplasty or vertebroplasties).mp. (4517)

24 (Viscosupplementation or viscosupplementations).mp. (717)

25 ((back or spine or spinal or cervical or lumbar or vertebra* or neck or hand or knee or hip or muscle or musculoskeletal or carpal tunnel or orthoped* or orthopaed*) adj3 (surgery or surgeries or surgical or procedure*)).mp. [mp=title, book title, abstract, original title, name of substance word, subject heading word, floating sub-heading word, keyword heading word, organism supplementary concept word, protocol supplementary concept word, rare disease supplementary concept word, unique identifier, synonyms, population supplementary concept word, anatomy supplementary concept word] (139028)

26 or/1-25 (515143)

27 Pain, Postoperative/ (47564)

28 ((postoperative adj6 pain*) or (post-operative adj6 pain*) or post-operative-pain*).mp. [mp=title, book title, abstract, original title, name of substance word, subject heading word, floating sub-heading word, keyword heading word, organism supplementary concept word, protocol supplementary concept word, rare disease supplementary concept word, unique identifier, synonyms, population supplementary concept word, anatomy supplementary concept word] (76169)

29 ((post-operative adj6 analgesi*) or (postoperative adj6 analgesi*)).mp. [mp=title, book title, abstract, original title, name of substance word, subject heading word, floating sub-heading word, keyword heading word, organism supplementary concept word, protocol supplementary concept word, rare disease supplementary concept word, unique identifier, synonyms, population supplementary concept word, anatomy supplementary concept word] (18600)

30 ((post-surgical adj6 pain*) or (post surgical adj6 pain*) or (post-surgery adj6 pain*) or (post adj surg* adj pain*)).mp. [mp=title, book title, abstract, original title, name of substance word, subject heading word, floating sub-heading word, keyword heading word, organism supplementary concept word, protocol supplementary concept word, rare disease supplementary concept word, unique identifier, synonyms, population supplementary concept word, anatomy supplementary concept word] (1270)

31 ((post* adj pain*) or pain relief after or pain following surg*).mp. [mp=title, book title, abstract, original title, name of substance word, subject heading word, floating sub-heading word, keyword heading word, organism supplementary concept word, protocol supplementary concept word, rare disease supplementary concept word, unique identifier, synonyms, population supplementary concept word, anatomy supplementary concept word] (37953)

32 ((posttreatment adj6 pain*) or (pain control after adj6 surg*) or (post-surg* and (pain* or discomfort))).mp. [mp=title, book title, abstract, original title, name of substance word, subject heading word, floating sub-heading word, keyword heading word, organism supplementary concept word, protocol supplementary concept word, rare disease supplementary concept word, unique identifier, synonyms, population supplementary concept word, anatomy supplementary concept word] (4775)

33 ((analgesi* adj6 postoperat*) or (analgesi* adj6 post-operat*) or (pain* adj6 after surg*) or (pain* adj6 after operat*) or (analgesi* adj6 after operat*)).mp. [mp=title, book title, abstract, original title, name of substance word, subject heading word, floating sub-heading word, keyword heading word, organism supplementary concept word, protocol supplementary concept word, rare disease supplementary concept word, unique identifier, synonyms, population supplementary concept word, anatomy supplementary concept word] (26278)

34 ((pain* or analgesi*) adj6 ("follow* operat*" or "follow* surg*")).mp. [mp=title, book title, abstract, original title, name of substance word, subject heading word, floating sub-heading word, keyword heading word, organism supplementary concept word, protocol supplementary concept word, rare disease supplementary concept word, unique identifier, synonyms, population supplementary concept word, anatomy supplementary concept word] (1085)

35 (pain* or discomfort or analgesi*).mp. [mp=title, book title, abstract, original title, name of substance word, subject heading word, floating sub-heading word, keyword heading word, organism supplementary concept word, protocol supplementary concept word, rare disease supplementary concept word, unique identifier, synonyms, population supplementary concept word, anatomy supplementary concept word] (1067518)

36 (chronic* or constant* or continu* or persist* or longterm or long-term or longstanding or long-standing or long lasting or long-lasting or phantom or sustain* or prolong* or recurr*).mp. [mp=title, book title, abstract, original title, name of substance word, subject heading word, floating sub-heading word, keyword heading word, organism supplementary concept word, protocol supplementary concept word, rare disease supplementary concept word, unique identifier, synonyms, population supplementary concept word, anatomy supplementary concept word] (5794751)

37 or/27-34 (91394)

38 (chronic* or constant* or continu* or persist* or longterm or long-term or longstanding or long-standing or long lasting or long-lasting or phantom or sustain* or prolong* or recurr*).mp. [mp=title, book title, abstract, original title, name of substance word, subject heading word, floating sub-heading word, keyword heading word, organism supplementary concept word, protocol supplementary concept word, rare disease supplementary concept word, unique identifier, synonyms, population supplementary concept word, anatomy supplementary concept word] (5794751)

39 (preoperative or pre-operative).mp. [mp=title, book title, abstract, original title, name of substance word, subject heading word, floating sub-heading word, keyword heading word, organism supplementary concept word, protocol supplementary concept word, rare disease supplementary concept word, unique identifier, synonyms, population supplementary concept word, anatomy supplementary concept word] (370217)

40 (peri-operative or perioperative).mp. [mp=title, book title, abstract, original title, name of substance word, subject heading word, floating sub-heading word, keyword heading word, organism supplementary concept word, protocol supplementary concept word, rare disease supplementary concept word, unique identifier, synonyms, population supplementary concept word, anatomy supplementary concept word] (138836)

41 38 or 39 or 40 (6123231)

42 26 and 37 and 41 (12869)

43 randomized controlled trial.pt. (591388)

44 controlled clinical trial.pt. (95278)

45 randomi?ed.ab. (717304)

46 placebo.ab. (237602)

47 drug therapy.fs. (2584307)

48 randomly.ab. (406741)

49 trial.ab. (645213)

50 groups.ab. (2507140)

51 or/43-50 (5658282)

52 exp animals/ not humans.sh. (5115172)

53 51 not 52 (4938130)

54 42 and 53 (6327)

**Embase (OVID)**

Search Strategy:

--------------------------------------------------------------------------------

1 exp orthopedic surgery/ (581449)

2 (orthop?edic* adj2 (procedure* or surgery or surgical or surgeries)).mp. [mp=title, abstract, heading word, drug trade name, original title, device manufacturer, drug manufacturer, device trade name, keyword heading word, floating subheading word, candidate term word] (59585)

3 (Acetabuloplasty or acetabuloplasties).mp. [mp=title, abstract, heading word, drug trade name, original title, device manufacturer, drug manufacturer, device trade name, keyword heading word, floating subheading word, candidate term word] (1602)

4 (((amputation or amputations) adj2 surgical) or (disarticulation or hemipelvecomy)).mp. [mp=title, abstract, heading word, drug trade name, original title, device manufacturer, drug manufacturer, device trade name, keyword heading word, floating subheading word, candidate term word] (2310)

5 (Arthrodesis or arthrodeses or spinal fusion* or lumbar fusion* or cervical fusion* or spondylodeses or spondylodesis or spondylosyndeses or spondylosyndesis).mp. [mp=title, abstract, heading word, drug trade name, original title, device manufacturer, drug manufacturer, device trade name, keyword heading word, floating subheading word, candidate term word] (41338)

6 (Arthroplasty or arthroplasties or (bone tunnel adj (enlargement or enlargements or widening))).mp. (128762)

7 (Arthroscopy or arthroscopic).mp. [mp=title, abstract, heading word, drug trade name, original title, device manufacturer, drug manufacturer, device trade name, keyword heading word, floating subheading word, candidate term word] (59569)

8 Bone lengthening.mp. (668)

9 (Bone transplantation or bone grafting).mp. [mp=title, abstract, heading word, drug trade name, original title, device manufacturer, drug manufacturer, device trade name, keyword heading word, floating subheading word, candidate term word] (28001)

10 (cementoplasty or cementoplasties or osteoplasties or osteoplasty).mp. [mp=title, abstract, heading word, drug trade name, original title, device manufacturer, drug manufacturer, device trade name, keyword heading word, floating subheading word, candidate term word] (1623)

11 ((Collateral or cruciate) adj ligament reconstruction).mp. [mp=title, abstract, heading word, drug trade name, original title, device manufacturer, drug manufacturer, device trade name, keyword heading word, floating subheading word, candidate term word] (19826)

12 (diskectomy or diskectomies or discectomy or discectomies).mp. [mp=title, abstract, heading word, drug trade name, original title, device manufacturer, drug manufacturer, device trade name, keyword heading word, floating subheading word, candidate term word] (17626)

13 fasciotomy/ (6180)

14 (fascietomy or fasciotomy).mp. [mp=title, abstract, heading word, drug trade name, original title, device manufacturer, drug manufacturer, device trade name, keyword heading word, floating subheading word, candidate term word] (7167)

15 ((fracture or skeletal) adj (surgery or fixation*)).mp. [mp=title, abstract, heading word, drug trade name, original title, device manufacturer, drug manufacturer, device trade name, keyword heading word, floating subheading word, candidate term word] (33350)

16 ((Disc or disk) adj (Chemolysis or chemolyses or nucleolyses or nucleolysis)).mp. [mp=title, abstract, heading word, drug trade name, original title, device manufacturer, drug manufacturer, device trade name, keyword heading word, floating subheading word, candidate term word] (9)

17 (Laminectomy or laminectomies or laminotomies or laminotomy).mp. [mp=title, abstract, heading word, drug trade name, original title, device manufacturer, drug manufacturer, device trade name, keyword heading word, floating subheading word, candidate term word] (29420)

18 (Laminoplasty or laminaplasties or laminaplasty or laminoplasties).mp. [mp=title, abstract, heading word, drug trade name, original title, device manufacturer, drug manufacturer, device trade name, keyword heading word, floating subheading word, candidate term word] (3757)

19 Limb Salvage.mp. or limb salvage/ (13950)

20 (Osteotomy or osteotomies).mp. [mp=title, abstract, heading word, drug trade name, original title, device manufacturer, drug manufacturer, device trade name, keyword heading word, floating subheading word, candidate term word] (62267)

21 (Synovectomy or synovectomies or synovium resection*).mp. [mp=title, abstract, heading word, drug trade name, original title, device manufacturer, drug manufacturer, device trade name, keyword heading word, floating subheading word, candidate term word] (6421)

22 tendon transfer/ or Tendon transfer*.mp. (5584)

23 (Tenodesis or tenodeses).mp. [mp=title, abstract, heading word, drug trade name, original title, device manufacturer, drug manufacturer, device trade name, keyword heading word, floating subheading word, candidate term word] (3248)

24 (Tenotomy or ((heel cord or heel-cord or tendon) adj (release or releases or lengthening or lengthenings))).mp. [mp=title, abstract, heading word, drug trade name, original title, device manufacturer, drug manufacturer, device trade name, keyword heading word, floating subheading word, candidate term word] (5828)

25 (vertebroplasty or vertebroplasties).mp. [mp=title, abstract, heading word, drug trade name, original title, device manufacturer, drug manufacturer, device trade name, keyword heading word, floating subheading word, candidate term word] (7429)

26 (Viscosupplementation or viscosupplementations).mp. [mp=title, abstract, heading word, drug trade name, original title, device manufacturer, drug manufacturer, device trade name, keyword heading word, floating subheading word, candidate term word] (1168)

27 ((back or spine or spinal or cervical or lumbar or vertebra* or neck or hand or knee or hip or muscle or musculoskeletal or carpal tunnel or orthoped* or orthopaed*) adj3 (surgery or surgeries or surgical or procedure*)).mp. [mp=title, abstract, heading word, drug trade name, original title, device manufacturer, drug manufacturer, device trade name, keyword heading word, floating subheading word, candidate term word] (222697)

28 postoperative pain/ (86559)

29 ((postoperative adj6 pain*) or (post-operative adj6 pain*) or post-operative-pain*).mp. [mp=title, abstract, heading word, drug trade name, original title, device manufacturer, drug manufacturer, device trade name, keyword heading word, floating subheading word, candidate term word] (120045)

30 ((post-operative adj6 analgesi*) or (postoperative adj6 analgesi*)).mp. [mp=title, abstract, heading word, drug trade name, original title, device manufacturer, drug manufacturer, device trade name, keyword heading word, floating subheading word, candidate term word] (39837)

31 ((post-surgical adj6 pain*) or (post surgical adj6 pain*) or (post-surgery adj6 pain*) or (post adj surg* adj pain*)).mp. [mp=title, abstract, heading word, drug trade name, original title, device manufacturer, drug manufacturer, device trade name, keyword heading word, floating subheading word, candidate term word] (2473)

32 ((post* adj pain*) or pain relief after or pain following surg*).mp. [mp=title, abstract, heading word, drug trade name, original title, device manufacturer, drug manufacturer, device trade name, keyword heading word, floating subheading word, candidate term word] (101838)

33 ((posttreatment adj6 pain*) or (pain control after adj6 surg*) or (post-surg* and (pain* or discomfort))).mp. [mp=title, abstract, heading word, drug trade name, original title, device manufacturer, drug manufacturer, device trade name, keyword heading word, floating subheading word, candidate term word] (9793)

34 ((analgesi* adj6 postoperat*) or (analgesi* adj6 post-operat*) or (pain* adj6 after surg*) or (pain* adj6 after operat*) or (analgesi* adj6 after operat*)).mp. [mp=title, abstract, heading word, drug trade name, original title, device manufacturer, drug manufacturer, device trade name, keyword heading word, floating subheading word, candidate term word] (50056)

35 ((pain* or analgesi*) adj6 ("follow* operat*" or "follow* surg*")).mp. [mp=title, abstract, heading word, drug trade name, original title, device manufacturer, drug manufacturer, device trade name, keyword heading word, floating subheading word, candidate term word] (1643)

36 or/1-27 (741989)

37 or/28-35 (149946)

38 36 and 37 (38991)

39 (chronic* or constant* or continu* or persist* or longterm or long-term or longstanding or long-standing or long lasting or long-lasting or phantom or sustain* or prolong* or recurr*).mp. [mp=title, abstract, heading word, drug trade name, original title, device manufacturer, drug manufacturer, device trade name, keyword heading word, floating subheading word, candidate term word] (8028276)

40 (preoperative or pre-operative).mp. [mp=title, abstract, heading word, drug trade name, original title, device manufacturer, drug manufacturer, device trade name, keyword heading word, floating subheading word, candidate term word] (591550)

41 (peri-operative or perioperative).mp. [mp=title, abstract, heading word, drug trade name, original title, device manufacturer, drug manufacturer, device trade name, keyword heading word, floating subheading word, candidate term word] (216487)

42 39 or 40 or 41 (8522949)

43 38 and 42 (22807)

44 randomized controlled trial/ (781096)

45 Controlled clinical study/ (469077)

46 random$.ti,ab. (1956864)

47 randomization/ (98871)

48 intermethod comparison/ (296379)

49 placebo.ti,ab. (364571)

50 (compare or compared or comparison).ti. (601559)

51 ((evaluated or evaluate or evaluating or assessed or assess) and (compare or compared or comparing or comparison)).ab. (2749062)

52 (open adj label).ti,ab. (108316)

53 ((double or single or doubly or singly) adj (blind or blinded or blindly)).ti,ab. (273374)

54 double blind procedure/ (209600)

55 parallel group$1.ti,ab. (32062)

56 (crossover or cross over).ti,ab. (124003)

57 ((assign$ or match or matched or allocation) adj5 (alternate or group$1 or intervention$1 or patient$1 or subject$1 or participant$1)).ti,ab. (412672)

58 (assigned or allocated).ti,ab. (486220)

59 (controlled adj7 (study or design or trial)).ti,ab. (448299)

60 (volunteer or volunteers).ti,ab. (281408)

61 human experiment/ (646707)

62 trial.ti. (400492)

63 or/44-62 (6276913)

64 (random$ adj sampl$ adj7 ("cross section$" or questionnaire$1 or survey$ or database$1)).ti,ab. not (comparative study/ or controlled study/ or randomi?ed controlled.ti,ab. or randomly assigned.ti,ab.) (9435)

65 Cross-sectional study/ not (randomized controlled trial/ or controlled clinical study/ or controlled study/ or randomi?ed controlled.ti,ab. or control group$1.ti,ab.) (345280)

66 (((case adj control$) and random$) not randomi?ed controlled).ti,ab. (21463)

67 (Systematic review not (trial or study)).ti. (257456)

68 (nonrandom$ not random$).ti,ab. (18853)

69 "Random field$".ti,ab. (2936)

70 (random cluster adj3 sampl$).ti,ab. (1530)

71 (review.ab. and review.pt.) not trial.ti. (1108620)

72 "we searched".ab. and (review.ti. or review.pt.) (49340)

73 "update review".ab. (138)

74 (databases adj4 searched).ab. (61639)

75 (rat or rats or mouse or mice or swine or porcine or murine or sheep or lambs or pigs or piglets or rabbit or rabbits or cat or cats or dog or dogs or cattle or bovine or monkey or monkeys or trout or marmoset$1).ti. and animal experiment/ (1222539)

76 Animal experiment/ not (human experiment/ or human/) (2567727)

77 or/64-76 (4308958)

78 63 not 77 (5541341)

79 43 and 78 (8744)

**Cochrane Library**

Search Strategy:

--------------------------------------------------------------------------------

ID Search Hits

#1 MeSH descriptor: [Orthopedic Procedures] explode all trees 17348

#2 (orthop?edic* NEAR/2 (procedure* or surgery or surgical or surgeries)) 10650

#3 Acetabuloplasty or acetabuloplasties 13

#4 (((amputation or amputations) NEAR/2 surgical) or (disarticulation or hemipelvectomy)) 656

#5 (Arthrodesis or arthrodeses or spinal fusion* or lumbar fusion* or cervical fusion* or spondylodeses or spondylodesis or spondylosyndeses or spondylosyndesis) 4456

#6 (Arthroplasty or arthroplasties or (bone tunnel NEAR (enlargement or enlargements or widening))) 15039

#7 Arthroscopy or arthroscopic 6558

#8 Bone lengthening 161

#9 Bone transplantation or bone grafting 9946

#10 cementoplasty or cementoplasties or osteoplasties or osteoplasty 52

#11 ((Collateral or cruciate) NEAR ligament reconstruction) 2605

#12 diskectomy or diskectomies or discectomy or discectomies 1823

#13 fascietomy or fasciotomy 234

#14 ((fracture or skeletal) NEAR (surgery or fixation*)) 6785

#15 (Intervertebral near (Disc or disk) near (Chemolysis or chemolyses or nucleolyses or nucleolysis)) 47

#16 Laminectomy or laminectomies or laminotomies or laminotomy 928

#17 Laminoplasty or laminaplasties or laminaplasty or laminoplasties 155

#18 Limb Salvage 559

#19 Osteotomy or osteotomies 2569

#20 Synovectomy or synovectomies or synovium resection* 157

#21 Tendon transfer* 276

#22 Tenodesis or tenodeses 226

#23 (Tenotomy or ((heel cord or heel-cord or tendon) NEAR (release or releases or lengthening or lengthenings))) 357

#24 vertebroplasty or vertebroplasties 440

#25 Viscosupplementation or viscosupplementations 210

#26 ((back or spine or spinal or cervical or lumbar or vertebra* or neck or hand or knee or hip or muscle or musculoskeletal or carpal tunnel or orthoped* or orthopaed*) adj3 (surgery or surgeries or surgical or procedure*)).mp 19573

#27 #1 or #2 or #3 or #4 or #5 or #6 or #7 or #8 or #9 or #10 or #11 or #12 or #13 or #14 or #15 or #16 or #17 or #18 or #19 or #20 or #21 or #22 or #23 or #24 or #25 or #26 72263

#28 MeSH descriptor: [Pain, Postoperative] explode all trees 18763

#29 ((postoperative NEAR/6 pain*) or (post-operative NEAR/6 pain*) or post-operative-pain*) 46110

#30 ((post-operative NEAR/6 analgesi*) or (postoperative NEAR/6 analgesi*)) 20823

#31 ((post-surgical NEAR/6 pain*) or (post surgical NEAR/6 pain*) or (post-surgery NEAR/6 pain*) or (post NEAR surg* NEAR pain*)) 3969

#32 ((post* NEAR pain*) or pain relief after or pain following surg*) 75802

#33 ((posttreatment NEAR/6 pain*) or (pain control after NEAR/6 surg*) or (post-surg* and (pain* or discomfort))) 17111

#34 ((analgesi* NEAR/6 postoperat*) or (analgesi* NEAR/6 post-operat*) or (pain* NEAR/6 after surg*) or (pain* NEAR/6 after operat*) or (analgesi* NEAR/6 after operat*)) 37351

#35 ((pain* or analgesi*) NEAR/6 ("follow* operat*" or "follow* surg*")) 49

#36 #28 or #29 or #30 or #31 or #32 or #33 or #34 or #35 87508

#37 #27 AND #36 16317

#38 chronic* or constant* or continu* or persist* or longterm or long-term or longstanding or long-standing or long lasting or long-lasting or phantom or sustain* or prolong* or recurr* 562704

#39 preoperative or pre-operative 45013

#40 peri-operative or perioperative 25436

#41 #38 or #39 or #40 607312

#42 #37 and #41 in Trials 7182

**PsycInfo (OVID)**

**Database: APA PsycInfo**

Search Strategy:

--------------------------------------------------------------------------------

1 (orthop?edic* adj2 (procedure* or surgery or surgical or surgeries)).mp. [mp=title, abstract, heading word, table of contents, key concepts, original title, tests & measures, mesh word] (728)

2 (Acetabuloplasty or acetabuloplasties).mp. [mp=title, abstract, heading word, table of contents, key concepts, original title, tests & measures, mesh word] (0)

3 (((amputation or amputations) adj2 surgical) or (disarticulation or hemipelvectomy)).mp. [mp=title, abstract, heading word, table of contents, key concepts, original title, tests & measures, mesh word] (99)

4 (Arthrodesis or arthrodeses or spinal fusion* or lumbar fusion* or cervical fusion* or spondylodeses or spondylodesis or spondylosyndeses or spondylosyndesis).mp. [mp=title, abstract, heading word, table of contents, key concepts, original title, tests & measures, mesh word] (172)

5 (Arthroplasty or arthroplasties or (bone tunnel adj (enlargement or enlargements or widening))).mp. [mp=title, abstract, heading word, table of contents, key concepts, original title, tests & measures, mesh word] (845)

6 (Arthroscopy or arthroscopic).mp. [mp=title, abstract, heading word, table of contents, key concepts, original title, tests & measures, mesh word] (100)

7 Bone lengthening.mp. (5)

8 (Bone transplantation or bone grafting).mp. [mp=title, abstract, heading word, table of contents, key concepts, original title, tests & measures, mesh word] (14)

9 (cementoplasty or cementoplasties or osteoplasties or osteoplasty).mp. [mp=title, abstract, heading word, table of contents, key concepts, original title, tests & measures, mesh word] (8)

10 ((Collateral or cruciate) adj ligament reconstruction).mp. [mp=title, abstract, heading word, table of contents, key concepts, original title, tests & measures, mesh word] (95)

11 (diskectomy or diskectomies or discectomy or discectomies).mp. [mp=title, abstract, heading word, table of contents, key concepts, original title, tests & measures, mesh word] (114)

12 (fascietomy or fasciotomy).mp. [mp=title, abstract, heading word, table of contents, key concepts, original title, tests & measures, mesh word] (8)

13 ((fracture or skeletal) adj (surgery or fixation*)).mp. [mp=title, abstract, heading word, table of contents, key concepts, original title, tests & measures, mesh word] (171)

14 (Intervertebral adj (Disc or disk) adj (Chemolysis or chemolyses or nucleolyses or nucleolysis)).mp. [mp=title, abstract, heading word, table of contents, key concepts, original title, tests & measures, mesh word] (0)

15 (Laminectomy or laminectomies or laminotomies or laminotomy).mp. [mp=title, abstract, heading word, table of contents, key concepts, original title, tests & measures, mesh word] (301)

16 (Laminoplasty or laminaplasties or laminaplasty or laminoplasties).mp. [mp=title, abstract, heading word, table of contents, key concepts, original title, tests & measures, mesh word] (9)

17 Limb Salvage.mp. (31)

18 (Osteotomy or osteotomies).mp. [mp=title, abstract, heading word, table of contents, key concepts, original title, tests & measures, mesh word] (57)

19 (Synovectomy or synovectomies or synovium resection*).mp. [mp=title, abstract, heading word, table of contents, key concepts, original title, tests & measures, mesh word] (1)

20 Tendon transfer*.mp. (13)

21 (Tenodesis or tenodeses).mp. [mp=title, abstract, heading word, table of contents, key concepts, original title, tests & measures, mesh word] (8)

22 (Tenotomy or ((heel cord or heel-cord or tendon) adj (release or releases or lengthening or lengthenings))).mp. [mp=title, abstract, heading word, table of contents, key concepts, original title, tests & measures, mesh word] (32)

23 (vertebroplasty or vertebroplasties).mp. [mp=title, abstract, heading word, table of contents, key concepts, original title, tests & measures, mesh word] (44)

24 (Viscosupplementation or viscosupplementations).mp. [mp=title, abstract, heading word, table of contents, key concepts, original title, tests & measures, mesh word] (6)

25 ((back or spine or spinal or cervical or lumbar or vertebra* or neck or hand or knee or hip or muscle or musculoskeletal or carpal tunnel or orthoped* or orthopaed*) adj3 (surgery or surgeries or surgical or procedure*)).mp. [mp=title, abstract, heading word, table of contents, key concepts, original title, tests & measures, mesh word] (2960)

26 or/1-25 (4416)

27 ((postoperative adj6 pain*) or (post-operative adj6 pain*) or post-operative-pain*).mp. [mp=title, abstract, heading word, table of contents, key concepts, original title, tests & measures, mesh word] (2663)

28 ((post-operative adj6 analgesi*) or (postoperative adj6 analgesi*)).mp. [mp=title, abstract, heading word, table of contents, key concepts, original title, tests & measures, mesh word] (555)

29 ((post-surgical adj6 pain*) or (post surgical adj6 pain*) or (post-surgery adj6 pain*) or (post adj surg* adj pain*)).mp. [mp=title, abstract, heading word, table of contents, key concepts, original title, tests & measures, mesh word] (196)

30 ((post* adj pain*) or pain relief after or pain following surg*).mp. [mp=title, abstract, heading word, table of contents, key concepts, original title, tests & measures, mesh word] (2210)

31 ((posttreatment adj6 pain*) or (pain control after adj6 surg*) or (post-surg* and (pain* or discomfort))).mp. [mp=title, abstract, heading word, table of contents, key concepts, original title, tests & measures, mesh word] (571)

32 ((analgesi* adj6 postoperat*) or (analgesi* adj6 post-operat*) or (pain* adj6 after surg*) or (pain* adj6 after operat*) or (analgesi* adj6 after operat*)).mp. [mp=title, abstract, heading word, table of contents, key concepts, original title, tests & measures, mesh word] (985)

33 ((pain* or analgesi*) adj6 ("follow* operat*" or "follow* surg*")).mp. [mp=title, abstract, heading word, table of contents, key concepts, original title, tests & measures, mesh word] (137)

34 (chronic* or constant* or continu* or persist* or longterm or long-term or longstanding or long-standing or long lasting or long-lasting or phantom or sustain* or prolong* or recurr*).mp. [mp=title, abstract, heading word, table of contents, key concepts, original title, tests & measures, mesh word] (882854)

35 (chronic* or constant* or continu* or persist* or longterm or long-term or longstanding or long-standing or long lasting or long-lasting or phantom or sustain* or prolong* or recurr*).mp. [mp=title, abstract, heading word, table of contents, key concepts, original title, tests & measures, mesh word] (882854)

36 (preoperative or pre-operative).mp. [mp=title, abstract, heading word, table of contents, key concepts, original title, tests & measures, mesh word] (5588)

37 (peri-operative or perioperative).mp. [mp=title, abstract, heading word, table of contents, key concepts, original title, tests & measures, mesh word] (1657)

38 35 or 36 or 37 (887800)

39 or/27-33 (3890)

40 26 and 39 (685)

41 38 and 40 (393)

**CINAHL**

Search Strategy:

--------------------------------------------------------------------------------

| **#** | **Query** | **Limiters/Expanders** | **Results** |
| --- | --- | --- | --- |
| S65 | S23 AND S64 | Search modes - Boolean/Phrase | 2,462 |
| S64 | S59 AND S63 | Search modes - Boolean/Phrase | 6,439 |
| S63 | S60 OR S61 OR S62 | Search modes - Boolean/Phrase | 1,537,436 |
| S62 | TX peri-operative or perioperative | Search modes - Boolean/Phrase | 108,178 |
| S61 | TX preoperative or pre-operative | Search modes - Boolean/Phrase | 83,816 |
| S60 | TX chronic* or constant* or continu* or persist* or longterm or long-term or longstanding or long-standing or long lasting or long-lasting or phantom or sustain* or prolong* or recurr* | Search modes - Boolean/Phrase | 1,401,440 |
| S59 | S49 AND S58 | Search modes - Boolean/Phrase | 11,565 |
| S58 | S50 OR S51 OR S52 OR S53 OR S54 OR S55 OR S56 OR S57 | Search modes - Boolean/Phrase | 34,959 |
| S57 | TX ((pain* or analgesi*) N6 ("follow* operat*" or "follow* surg*")) | Search modes - Boolean/Phrase | 461 |
| S56 | TX ((analgesi* N6 postoperat*) or (analgesi* N6 post-operat*) or (pain* N6 after surg*) or (pain* N6 after operat*) or (analgesi* N6 after operat*)) | Search modes - Boolean/Phrase | 7,292 |
| S55 | TX ((posttreatment N6 pain*) or (pain control after N6 surg*) or (post-surg* and (pain* or discomfort))) | Search modes - Boolean/Phrase | 1,940 |
| S54 | TX ((post* N1 pain*) or pain relief after or pain following surg*) | Search modes - Boolean/Phrase | 29,808 |
| S53 | TX ((post-surgical N6 pain*) or (post surgical N6 pain*) or (post-surgery N6 pain*) or (post adj surg* N1 pain*)) | Search modes - Boolean/Phrase | 573 |
| S52 | TX ((post-operative N6 analgesi*) or (postoperative N6 analgesi*)) | Search modes - Boolean/Phrase | 5,157 |
| S51 | TX ((postoperative N6 pain*) or (post-operative N6 pain*) or post-operative-pain*) | Search modes - Boolean/Phrase | 27,432 |
| S50 | (MH "Postoperative Pain") | Search modes - Boolean/Phrase | 20,645 |
| S49 | S24 OR S25 OR S26 OR S27 OR S28 OR S29 OR S30 OR S31 OR S32 OR S33 OR S34 OR S35 OR S36 OR S37 OR S38 OR S39 OR S40 OR S41 OR S42 OR S43 OR S44 OR S45 OR S46 OR S47 OR S48 | Search modes - Boolean/Phrase | 309,950 |
| S48 | TX ((back or spine or spinal or cervical or lumbar or vertebra* or neck or hand or knee or hip or muscle or musculoskeletal or carpal tunnel or orthoped* or orthopaed*) N3 (surgery or surgeries or surgical or procedure*)) | Search modes - Boolean/Phrase | 233,351 |
| S47 | TX Viscosupplementation or viscosupplementations | Search modes - Boolean/Phrase | 243 |
| S46 | TX vertebroplasty or vertebroplasties | Search modes - Boolean/Phrase | 1,694 |
| S45 | TX (Tenotomy or ((heel cord or heel-cord or tendon) N1 (release or releases or lengthening or lengthenings))) | Search modes - Boolean/Phrase | 1,301 |
| S44 | TX Tenodesis or tenodeses | Search modes - Boolean/Phrase | 1,207 |
| S43 | TX Tendon transfer* | Search modes - Boolean/Phrase | 883 |
| S42 | TX (Synovectomy or synovectomies or synovium resection*) | Search modes - Boolean/Phrase | 463 |
| S41 | TX Osteotomy or osteotomies | Search modes - Boolean/Phrase | 13,461 |
| S40 | (MH "Limb Salvage") OR "Limb Salvage" | Search modes - Boolean/Phrase | 2,590 |
| S39 | TX Laminoplasty or laminaplasties or laminaplasty or laminoplasties | Search modes - Boolean/Phrase | 785 |
| S38 | TX Laminectomy or laminectomies or laminotomies or laminotomy | Search modes - Boolean/Phrase | 3,416 |
| S37 | TX (Intervertebral N1 (Disc or disk) N1 (Chemolysis or chemolyses or nucleolyses or nucleolysis)) | Search modes - Boolean/Phrase | 93 |
| S36 | TX ((fracture or skeletal) N1 (surgery or fixation*)) | Search modes - Boolean/Phrase | 34,972 |
| S35 | TX fascietomy or fasciotomy | Search modes - Boolean/Phrase | 1,090 |
| S34 | TX diskectomy or diskectomies or discectomy or discectomies | Search modes - Boolean/Phrase | 4,053 |
| S33 | TX ((Collateral or cruciate) N1 ligament reconstruction) | Search modes - Boolean/Phrase | 8,616 |
| S32 | TX cementoplasty or cementoplasties or osteoplasties or osteoplasty | Search modes - Boolean/Phrase | 404 |
| S31 | TX (Bone transplantation or bone grafting) | Search modes - Boolean/Phrase | 9,059 |
| S30 | TX Bone lengthening | Search modes - Boolean/Phrase | 1,028 |
| S29 | TX Arthroscopy or arthroscopic | Search modes - Boolean/Phrase | 29,365 |
| S28 | TX (Arthroplasty or arthroplasties or (bone tunnel N1 (enlargement or enlargements or widening))) | Search modes - Boolean/Phrase | 55,462 |
| S27 | TX Arthrodesis or arthrodeses or spinal fusion* or lumbar fusion* or cervical fusion* or spondylodeses or spondylodesis or spondylosyndeses or spondylosyndesis | Search modes - Boolean/Phrase | 17,846 |
| S26 | TX (((amputation or amputations) N2 surgical) or (disarticulation or hemipelvectomy)) | Search modes - Boolean/Phrase | 885 |
| S25 | TX Acetabuloplasty or acetabuloplasties | Search modes - Boolean/Phrase | 181 |
| S24 | (MH "Orthopedic Surgery+") | Search modes - Boolean/Phrase | 133,850 |
| S23 | S22 NOT S21 | Expanders - Apply equivalent subjects Search modes - Boolean/Phrase | 952,806 |
| S22 | S1 OR S2 OR S3 OR S4 OR S5 OR S6 OR S7 OR S8 OR S9 OR S10 OR S11 OR S12 OR S13 OR S14 OR S15 | Expanders - Apply equivalent subjects Search modes - Boolean/Phrase | 999,836 |
| S21 | S19 NOT S20 | Expanders - Apply equivalent subjects Search modes - Boolean/Phrase | 211,623 |
| S20 | MH (human) | Expanders - Apply equivalent subjects Search modes - Boolean/Phrase | 2,644,934 |
| S19 | S16 OR S17 OR S18 | Expanders - Apply equivalent subjects Search modes - Boolean/Phrase | 245,337 |
| S18 | TI (animal model*) | Expanders - Apply equivalent subjects Search modes - Boolean/Phrase | 3,471 |
| S17 | MH (animal studies) | Expanders - Apply equivalent subjects Search modes - Boolean/Phrase | 150,981 |
| S16 | MH animals+ | Expanders - Apply equivalent subjects Search modes - Boolean/Phrase | 103,326 |
| S15 | AB (cluster W3 RCT) | Expanders - Apply equivalent subjects Search modes - Boolean/Phrase | 488 |
| S14 | MH (crossover design) OR MH (comparative studies) | Expanders - Apply equivalent subjects Search modes - Boolean/Phrase | 470,674 |
| S13 | AB (control W5 group) | Expanders - Apply equivalent subjects Search modes - Boolean/Phrase | 142,419 |
| S12 | PT (randomized controlled trial) | Expanders - Apply equivalent subjects Search modes - Boolean/Phrase | 149,178 |
| S11 | MH (placebos) | Expanders - Apply equivalent subjects Search modes - Boolean/Phrase | 13,586 |
| S10 | MH (sample size) AND AB (assigned OR allocated OR control) | Expanders - Apply equivalent subjects Search modes - Boolean/Phrase | 4,380 |
| S9 | TI (trial) | Expanders - Apply equivalent subjects Search modes - Boolean/Phrase | 177,672 |
| S8 | AB (random*) | Expanders - Apply equivalent subjects Search modes - Boolean/Phrase | 394,773 |
| S7 | TI (randomised OR randomized) | Expanders - Apply equivalent subjects Search modes - Boolean/Phrase | 138,425 |
| S6 | MH cluster sample | Expanders - Apply equivalent subjects Search modes - Boolean/Phrase | 5,093 |
| S5 | MH pretest-posttest design | Expanders - Apply equivalent subjects Search modes - Boolean/Phrase | 51,721 |
| S4 | MH random assignment | Expanders - Apply equivalent subjects Search modes - Boolean/Phrase | 78,129 |
| S3 | MH single-blind studies | Expanders - Apply equivalent subjects Search modes - Boolean/Phrase | 15,879 |
| S2 | MH double-blind studies | Expanders - Apply equivalent subjects Search modes - Boolean/Phrase | 53,804 |
| S1 | MH randomized controlled trials | Expanders - Apply equivalent subjects Search modes - Boolean/Phrase | 135,417 |
